# Supplementary material for: BMI Modifies Increased Mortality Risk of Post-PCI STEMI Patients with AKI
Source: J Clin Med. 2022 Oct 17;11(20):6104. doi: 10.3390/jcm11206104 (PMC9604849; doi:10.3390/jcm11206104)
Supplement: Supplementary file 1 [file jcm-11-06104-s001.zip › jcm-1931998-supplementary.pdf]

Table S1- Baseline characteristics of patients with and without recorded BMI

	BMI not recorded	N	BMI recorded	N	pV
Age	62.1 ± 13.1	519	61.6 ± 13.1	1604	0.41
Gender (F)	22.4%	519	18.9%	1604	0.08
Smoker	45.9%	519	52%	1604	0.48
DM	23.3%	519	23.4%	1604	0.97
HTN	47.4%	519	42.5%	1604	0.051
HPL	49.1%	519	46.4%	1604	0.275
Past MI	9.4%	519	14.6%	1604	0.003
Cr (mg/dL)	1.15 ± 0.2	516	1.11 ± 0.3	1601	0.01
CRP (mg/L)	11.6 (1.-12.6)	158	13.4 (1.5-10.7)	1202	0.186
CPK (U/L)	1172 (291-1607)	504	1353 (340-1762)	1579	0.02
Hb (g/dL)	14.3 ± 1.5	517	14.2 ± 1.6	1602	0.287
Time to reperfusion (min)	378 (112-420)	517	403 (105-405)	1576	0.91
Contrast volume (ml)	141.1 (101-170)	38	146.1 (115-174)	380	0.39
Hemodynamic instability*	6%	519	4.1%	1603	0.07
CAD	0-0.2%, 1-42.7%, 2- 30.2%, 3+26.9%	513	3-0.1%, 1-42.5%, 2- 29.1%, 3+28.3%	1590	0.9
EF (%)	48.9% (40-55)	499	46.6% (40-50)	1585	<0.001
AKI	10.0%	499	10.6%	1207	0.71
Follow up time	46.6 ± 29.8	511	49.2 ± 30.5	1577	0.09

*Values are mean ±SD, n (%), or median (interquartile range). *Hemodynamic instability was recorded as patients' need for inotropes or intra-aortic balloon.*

CAD= Number of vessels with significant coronary artery disease; CPK= Creatine phosphokinase; Cr= Creatinine on admission; CRP= C reactive protein; DM= Diabetes Mellitus; EF= Ejection fraction; Hb= Hemoglobin; HPL= Hyperlipidemia; HTN= Hypertension; min= Minutes; wks= Weeks; yrs= Years.

Table S2- Baseline characteristics of patients with and without recorded AKI

	AKI not recorded	N	AKI recorded	N	pV
Age	62.7 ± 13.9	417	61.4 ± 12.9	1706	0.08
Gender (F)	20.4%	417	19.6%	1706	0.71
BMI	26.9 ± 4.2	400	26.8 ± 4.0	1223	0.95
Smoker	52%	417	50.1%	1706	0.48
DM	30%	417	21.7%	1706	<0.001
HTN	46.5%	417	43%	1706	0.197
HPL	47%	417	47.1%	1706	0.98
Past MI	23.3%	417	10.9%	1706	<0.001
Cr (mg/dL)	1.05 ± 0.5	414	1.14 ± 0.2	1705	<0.001
CRP (mg/L)	14.9 (1.8-10.5)	409	12.4 (1.4-11.2)	951	0.14
CPK (U/L)	1427 (395-1824)	414	1280 (317-1649)	1669	0.005
Hb (g/dL)	14.0 ± 1.7	415	14.3 ± 1.5	1704	<0.001
Time to reperfusion (min)	462 (104-450)	388	382 (105-405)	1705	0.731
Contrast volume (ml)		0		418	
Hemodynamic instability*	7.9%	417	3.8%	1705	<0.001
CAD	0-0%, 1- 35.2%, 2- 28.7%, 3+36.2%	401	3-0.2%, 1- 44.2%, 2- 29.6%, 3+26%	1702	<0.001
EF (%)	45.9% (40-50)	413	47% (40-55)	1681	0.002
Follow up time	49.9 ± 31.1	413	48.3 ± 30.1	1675	0.33

*Values are mean ±SD, n (%), or median (interquartile range). *Hemodynamic instability was recorded as patients' need for inotropes or intra-aortic balloon.*

CAD= Number of vessels with significant coronary artery disease; CPK= Creatine phosphokinase; Cr= Creatinine on admission; CRP= C reactive protein; DM= Diabetes Mellitus; EF= Ejection fraction; Hb= Hemoglobin; HPL= Hyperlipidemia; HTN= Hypertension; min= Minutes; wks= Weeks; yrs= Years.

Table S3- BMI Class frequencies

	Cohort	With AKI	Without AKI
<18.5	14 (0.7%)	1 (0.8%)	12 (1.1%)
18.6-24.9	546 (25.5%)	41 (32%)	375 (34.8%)
25-29.9	730 (34.1%)	59 (46.1%)	478 (44.3%)
30-39.9	303 (14.2%)	27 (21.1%)	207 (19.2%)
>40	12 (0.6%)	0	7 (0.6%)
